# A Novel Rotation Scheme for MEMS IMU Error Mitigation Based on a Missile-Borne Rotation Semi-Strapdown Inertial Navigation System

**DOI:** 10.3390/s19071683

**Published:** 2019-04-09

**Authors:** Zhengyao Jing, Jie Li, Xi Zhang, Kaiqiang Feng, Tao Zheng

**Affiliations:** 1 Key Laboratory of Instrumentation Science & Dynamic Measurement, Ministry of Education, North University of China, Taiyuan 030051, China; s1706154@163.com (Z.J.); Zhangxi@nuc.edu.cn (X.Z.); b1506011@st.nuc.edu.cn (K.F.); 2 National Key Laboratory for Electronic Measurement Technology, North University of China, Taiyuan 030051, China; 3 Key Laboratory of Biomimetic Robots and Systems, Beijing Institute of Technology, Ministry of Education, Beijing 100081, China; 18634409496@163.com

**Keywords:** rotation semi-SINS, rotation scheme, rotating angular rate, error compensation

## Abstract

In previous research, a semi-strapdown inertial navigation system (SSINS), based on micro-electro-mechanical system (MEMS) sensors, was able to realize over-range measurement of the attitude information of high-rotation missiles by constructing a single axis “spin reduction” platform. However, the MEMS sensors in SSINS were corrupted by significant sensor errors. In order to further improve SSINS measurement accuracy, a rotational modulation technique has been introduced to compensate for sensor errors. The ideal modulation angular velocity is changed sharply to achieve a constant speed, while in practical applications, the angular rate of the rotating mechanism’s output needs to go through an acceleration-deceleration process. Furthermore, the stability of the modulation angular rate is difficult to achieve in a high-speed rotation environment. In this paper, a novel rotation scheme is proposed which can effectively suppress the residual error in the navigation coordinate system caused by the modulation angular rate error, including the acceleration-deceleration process and instability of angular rate. The experiment results show that the position and attitude accuracy of the new rotation scheme was increased by more than 56%. In addition, the proposed scheme is applicable to navigation accuracy improvement under various dynamic conditions.

## 1. Introduction

The high-precision measurement of high-speed rotation missile attitude information is a key technology of the guidance and precision strike, which is the main development trend of conventional high-speed rotation missile guidance [1,2]. Furthermore, the realization of accurate navigation and positioning for high-rotation missiles is a key technology that needs an urgent breakthrough [3,4]. In order to meet the practical requirements of low cost, small size, and micro-power consumption, a strapdown inertial navigation system (SINS) equipped with a micro-electro-mechanical system (MEMS) inertial sensor is usually used to measure the attitude of the missile independently, through the MEMS accelerometer and the MEMS gyroscope [5,6].

Firstly, the SINS is a traditional fixed connection structure which makes the MEMS sensors of the inertial navigation system inevitably sensitive to the effects of vibrations from the missile. Secondly, in the highly dynamic missile-borne environment, the missile’s angular rate in the roll axis is very large, resulting in the gyro measurement accuracy being sharply reduced. In an attempt to solve the above two problems and further realize high-precision measurement of the attitude of missiles, the concept of semi-strapdown INS (SSINS) was proposed by the Key Laboratory of Instrumentation Science and Dynamic Measurement [7,8]. In contrast to SINS, miniature inertial measurement unit (MIMU) in the SSINS is not rigidly attached to the missile but is connected to the carrier via the rotating mechanism, which provides a stable low-dynamic environment for SSINS and eliminates the interference of high-speed rotation with sensor accuracy. Therefore, gyros with small range can be used to measure missile attitude information in a relatively stable environment. For the above reasons, we chose SSINS in this work to measure the velocity, position and attitude information of the missile.

However, SSINS measurement accuracy is difficult to further improve, the main factor being that the MEMS sensors are corrupted by significant sensor errors. Much research has shown that rotation modulation technology is one effective way to compensate for the error of the inertial sensor’s constant drifts and some other constant or slowly varying errors [9,10,11,12].

The rotary INS technique has been widely applied in marine navigation for submarines and warships [13,14,15]. However, a few types of research have been conducted on rotary INS based on low-cost MEMS inertial measurement unit (IMU) in the high-rotation missile-borne environment, which has great potential applications in the near future and is the focus of this paper. Meanwhile, research on the rotation modulation scheme has also mainly focused on the low dynamic and long-haul application environment, and the modulation angular rate of the rotation scheme is set lower. On the contrary, the high-rotation and short-time characteristics of missile-borne environments make the modulation angular rate of the rotation scheme higher and thus the modulation angular rate error greater [16]. In SSINS, the rotary mechanism is required to follow the high-dynamic changes of the missile in real-time in a high-speed rotation environment, which inevitably causes problems such as overshoot and instability of the rotation angular rate, thus resulting in residual errors. Furthermore, a sudden change in angular rate further exacerbates the modulation angular rate error. Therefore, according to the main error characteristics of the rotating mechanism output, it is necessary to design a reasonable rotation scheme to offset the above error for the improvement of navigation accuracy. In this way, navigation precision can be improved greatly if an appropriate rotation scheme is adopted [17,18,19,20].

In recent years, improvement and design of rotary modulation schemes have attracted a lot of attention. A system should choose a suitable rotation scheme based on the error characteristics of its own. References [21,22] have analyzed the error characteristics of MIMU rotating around the *X*-axis, *Y*-axis, and *Z*-axis respectively, and have studied the error observability of the inertial navigation system rotating in different directions. This is instructive for knowing which axis the INS selects as the modulation axis. Sang et al. discovered the problem that system drift cannot be completely eliminated and that large error occurs without isolation of the carrier motion; for this reason, an improved four-position rotation scheme is proposed. This solution can offset the effect of carrier motion on system accuracy [23]. In Liu’s paper, three rotation schemes are proposed based on two-axis fiber rotary inertial navigation, and among these the third rotation scheme has an accuracy of 0.7 nmile/12 h [24]. However, some of the scale factor errors and installation errors in this scheme cannot be offset. In order to isolate the influence of carrier motion and modulate all constant error, Liu et al. analyzed and improved a three-axis rotation scheme based on a fiber optic gyroscope, and its navigation accuracy was improved, with the position error being 0.6 nmile/12 h [25]. Because the above rotation scheme was designed and improved for the ship-borne environment, it is not fully applicable to the missile-borne environment. Due to the small space of the missile-borne environment, it is not suitable for multi-axis rotation schemes, and it is also not suitable for installing high-precision motors with large volumes. The above rotation scheme did not consider the actual situation of the indexing mechanism, and simply analyzed the error suppression effect by using the idealized rotation method from the principle.

Work [26] has studied the influence of the acceleration-deceleration process of the modulation angular rate on the modulation effect of inertial sensors’ biases and calculated the optimal angular rate and acceleration. In work [27], Wang Tingjun analyzed the influence of acceleration and deceleration on the constant error of several traditional rotation schemes and proposed a 4-position reciprocating rotation scheme. This scheme considered the actual movement of the indexing mechanism. However, the angular rate instability error in the high-dynamic environment was not taken into account.

In this paper, according to the actual error characteristics of the SSINS, that is, the actual modulation angular rate output situation of the indexing mechanism under high rotation and a high dynamic environment, a new rotation scheme is designed. The new rotation scheme completely offsets the effect of acceleration-deceleration on constant drift and largely offsets the residual error caused by the instability modulation angular rate error. The effectiveness and practicability of the new rotation scheme are proved by an actual SSINS manufactured by our lab.

The remainder of this paper is organized as follows. Section 2 briefly illustrates the working principle of the SSINS and error modulation mechanism. In Section 3, the impact of acceleration-deceleration process and modulation angular rate error instability on navigation accuracy are analyzed. A new rotational modulation scheme is proposed and error suppression effectiveness is analyzed in Section 4. Section 5 discusses the innovations and shortcomings of the article. In Section 6, through contrast with other existing rotation schemes, the effectiveness of the new rotation scheme error suppression is verified in the actual system. Section 7 is the conclusion.

## 2. Rotary Semi-Strapdown Inertial Navigation System

### 2.1. Configuration and Working Principle of the SSINS

**Figure 1:**
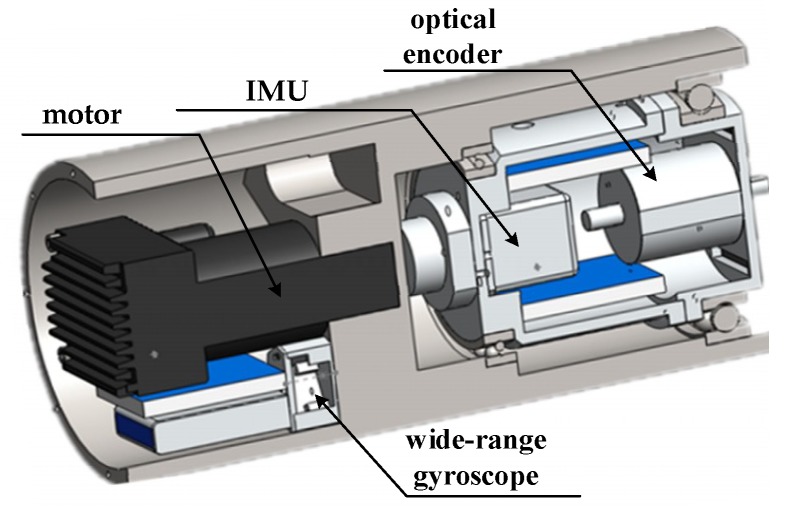
Main configuration of the semi-strapdown inertial navigation system (SSINS).

The SSINS used in this study, as shown in Figure 1, was mainly composed of an IMU, a motor, a wide-range gyroscope, an optical encoder, and control-related printed circuit boards (PCBs). The IMU mainly contained three small-range MEMS gyroscopes and three MEMS accelerometers.

**Figure 2:**
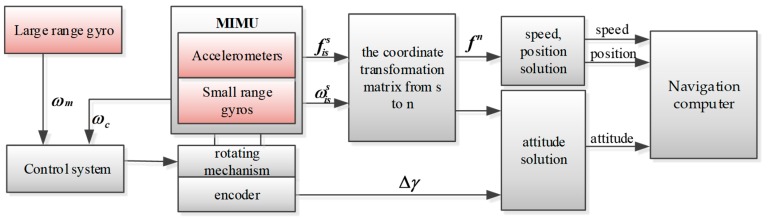
Working principle of the SSINS. Legend: MIMU, miniature inertial measurement unit.

In order to achieve high precision measurement of a high-speed rotation missile, in the first step, one wide-range gyro is mounted on the roll-axis of the missile and the speed of the missile ($\omega_{m}$) is measured by a wide-range gyroscope. Then, $\omega_{m}$ is fed back to the motor control module, so as to control the motor to drive the miniature inertial measurement unit (MIMU) to rotate in the opposite direction. Because of the limited precision of the wide-range gyroscope, the reversal of the missile’s rotational motion is incomplete. Hence, the axial angular rate changes from a high-speed rotation state to a low-speed rotation state. In the second step, the small-range gyro in the MIMU is used to measure the angular rate ($\omega_{c}$) that incompletely isolates the missile’s rolling movement, which is fed back to the control module to calculate the corrected angular rate (Δω) so as to reach the preset modulation angular rate ($\omega_{s}$) in real time. It can be seen from the above that the rotational modulation angular rate consists of two parts: the incompletely isolated missile’s rolling angular rate ($\omega_{c}$) and the corrected angular rate (Δω). The principle of the rotating semi-strapdown inertial navigation system (RSSINS) is shown in Figure 2.

### 2.2. Error Modulation Mechanism of the SSINS

#### 2.2.1. Definition of Coordinate System

**Figure 3:**
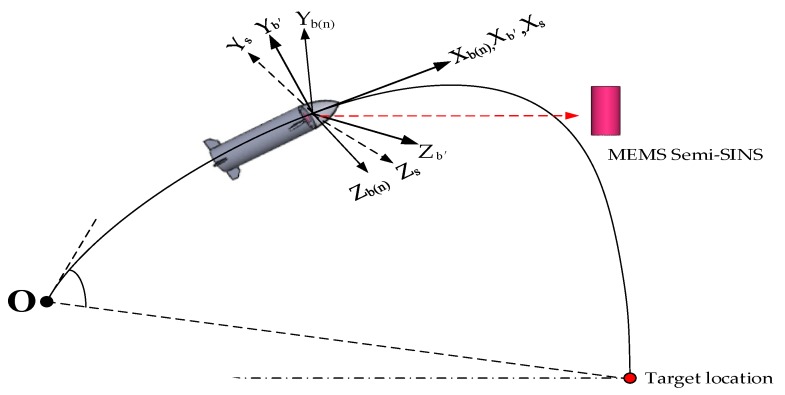
Definition of coordinate systems. Legend: MEMS, micro-electro-mechanical system.

In order to facilitate subsequent analysis, the missile-borne RSSINS’s coordinate systems can be seen in Figure 3. These systems are commonly used in this paper and are defined as follows: the navigation coordinate system (N-frame) is chosen as the local geographical coordinate frame and the body coordinate system (B-frame) is the missile’s coordinate system (we stipulate that the origin of its coordinates is at the missile’s center of mass, and that the $X_{b}$ axis, $Y_{b}$ axis, and $Z_{b}$ axis are the rolling axis, the yaw axis, and the pitch axis of the missile, respectively). In order to implement a rotation modulation method, a new frame in which the inertial readings are collected is introduced. The new coordinate system can be referred to as the inertial sensor frame (S-frame), and its axis is aligned with the sensitive axis of the inertial sensors. For the convenience of description, at the start, the S-frame is coincident with the B-frame. In addition, the B’-frame is defined as a virtual coordinate system and refers to B-frame before introduced the rotation modulation.

#### 2.2.2. Error Modulation Mechanism of the SSINS

We use a single-axis continuous rotation as the rotation method and take the zero-bias error of the gyro as an example with which to analyze the rotational modulation principle of the SSINS.

According to the working principle of SSINS in Section 2.1, we know that the rotational modulation angular rate ($\omega_{s}$) consists of two parts, these being the incompletely isolated missile’s rolling angular rate ($\omega_{c}$) and the corrected angular rate (Δω), that is:

(1) $$\omega_{s}=\Delta \omega +\omega_{c}$$

For the convenience of analysis, it is assumed here that $C_{b}^{n}$ = I. In SSINS, after conversion, the projection of the gyro drift in the navigation system is:

(2) $$\left[\begin{array}{c} \varepsilon_{E}\\ \varepsilon_{N}\\ \varepsilon_{U} \end{array}\right]=C_{b}^{n}C_{b\prime }^{b}C_{s}^{b\prime }\varepsilon^{s}=\left[\begin{array}{ccc} 1 & 0 & 0\\ 0 & \cos\omega_{c}t & -\sin\omega_{c}t\\ 0 & \sin\omega_{c}t & \cos\omega_{c}t \end{array}\right]\left[\begin{array}{ccc} 1 & 0 & 0\\ 0 & \cos\Delta \omega t & -\sin\Delta \omega t\\ 0 & \sin\Delta \omega t & \cos\Delta \omega t \end{array}\right]\left[\begin{array}{c} \varepsilon_{x}^{s}\\ \varepsilon_{y}^{s}\\ \varepsilon_{z}^{s} \end{array}\right]=\left[\begin{array}{ccc} 1 & 0 & 0\\ 0 & \cos\omega_{s}t & -\sin\omega_{s}t\\ 0 & \sin\omega_{s}t & \cos\omega_{s}t \end{array}\right]\left[\begin{array}{c} \varepsilon_{x}^{s}\\ \varepsilon_{y}^{s}\\ \varepsilon_{z}^{s} \end{array}\right]$$

The projection of the accelerometer bias under the navigation system is:

(3) $$\left[\begin{array}{c} \nabla_{E}\\ \nabla_{N}\\ \nabla_{U} \end{array}\right]=C_{b}^{n}C_{b\prime }^{b}C_{s}^{b\prime }\nabla^{s}=\left[\begin{array}{ccc} 1 & 0 & 0\\ 0 & \cos\omega_{c}t & -\sin\omega_{c}t\\ 0 & \sin\omega_{c}t & \cos\omega_{c}t \end{array}\right]\left[\begin{array}{ccc} 1 & 0 & 0\\ 0 & \cos\Delta \omega t & -\sin\Delta \omega t\\ 0 & \sin\Delta \omega t & \cos\Delta \omega t \end{array}\right]\left[\begin{array}{c} \nabla_{x}^{s}\\ \nabla_{y}^{s}\\ \nabla_{z}^{s} \end{array}\right]=\left[\begin{array}{ccc} 1 & 0 & 0\\ 0 & \cos\omega_{s}t & -\sin\omega_{s}t\\ 0 & \sin\omega_{s}t & \cos\omega_{s}t \end{array}\right]\left[\begin{array}{c} \nabla_{x}^{s}\\ \nabla_{y}^{s}\\ \nabla_{z}^{s} \end{array}\right]$$

According to Equations (2) and (3), the integration of gyro drifts and accelerometer bias in the navigation frame for a complete rotation cycle can be described by Equations (4) and (5):

(4) $$\int \varepsilon^{n}=\left[\begin{array}{c} T\varepsilon_{x}^{s}\\ 0\\ 0 \end{array}\right]$$

(5) $$\int \nabla^{n}=\left[\begin{array}{c} T\nabla_{x}^{s}\\ 0\\ 0 \end{array}\right]$$

It can be seen from Equations (4) and (5) that the rotational modulation modulates the zero-bias error of the *Y*- and *Z*-axis gyro and accelerometer into a sinusoidal form and integrates to zero in one rotation period. However, the error on the rotation axis is propagated according to the original law.

As we all know, the angular rate of the rotating mechanism output is a critical factor. Whether it is a continuous rotation or a transposition rotation scheme, only when the output of the motor is constant, the integral of $\omega_{s}t$ for one cycle of rotation is zero. Due to the dual effects of the hostile environment and actual output characteristics of the rotating mechanism, if $\omega_{s}$ is not constant, it is difficult to ensure that the integral of the inertial sensor’s constant drifts and some other constant or slowly varying errors after one cycle is zero.

## 3. Analysis Modulation Angular Rate Error of the Rotating Mechanism Output

In practical applications, the motor is required to rapidly respond in real-time to changes in the angular rate of the missile, and is affected by the high-dynamic environment, meaning that problems such as overshoot and instability of the angular rate are inevitable and that sensor errors cannot be completely suppressed and the performance of navigation accuracy is decreased. As we know, the stability and rapidity of control cannot be achieved at the same time. In the process control, in order to improve the response or tracking characteristics, when using overshoot to increase the speed of the control the motor will be shaken at the moment of starting, which causes the motor to start unsteadily. We can see that the output angular rate error occurring in the control process of the motor directly affects the stability of the rotational modulation angular rate, meaning that how to model the rotation of the motor is the premise for effectively analyzing the error modulation.

**Figure 4:**
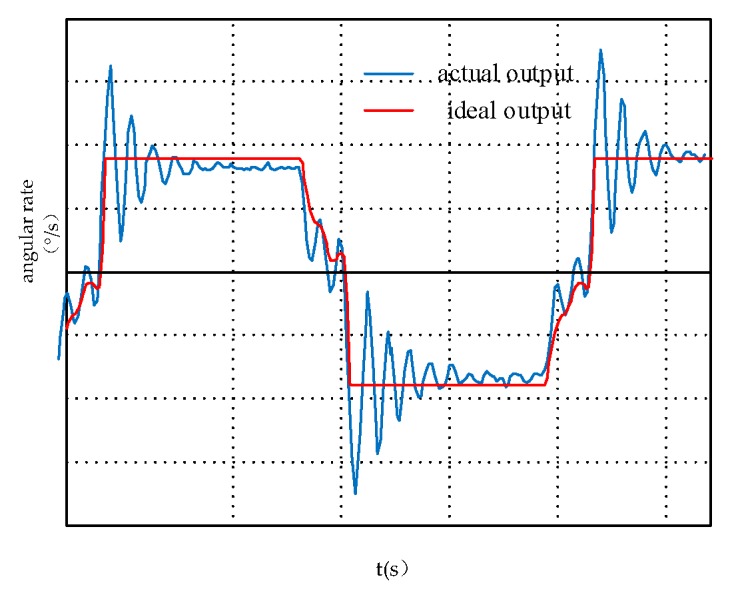
The ideal and actual output angular rate of the motor.

As shown in Figure 4, in the SSINS, the ideal and actual plot of the angular velocity of the motor output ranges from +ω to −ω. We can see that the acceleration-deceleration process and overshoot affect the symmetry of the angular rate. Therefore, the error modulation equation will have items related to acceleration-deceleration and overshoot.

In the SSINS, the errors of the gyros are the critical factors determining the accuracy of the system [28], and the error modulation of the accelerometer is similar to the gyroscope, so, taking the gyroscope as an example, the IMU rotates counterclockwise for one cycle and the influence of modulation angular rate error is analyzed in detail.

### 3.1. Modeling and Analysis of Motor Acceleration and Deceleration Process

**Figure 5:**
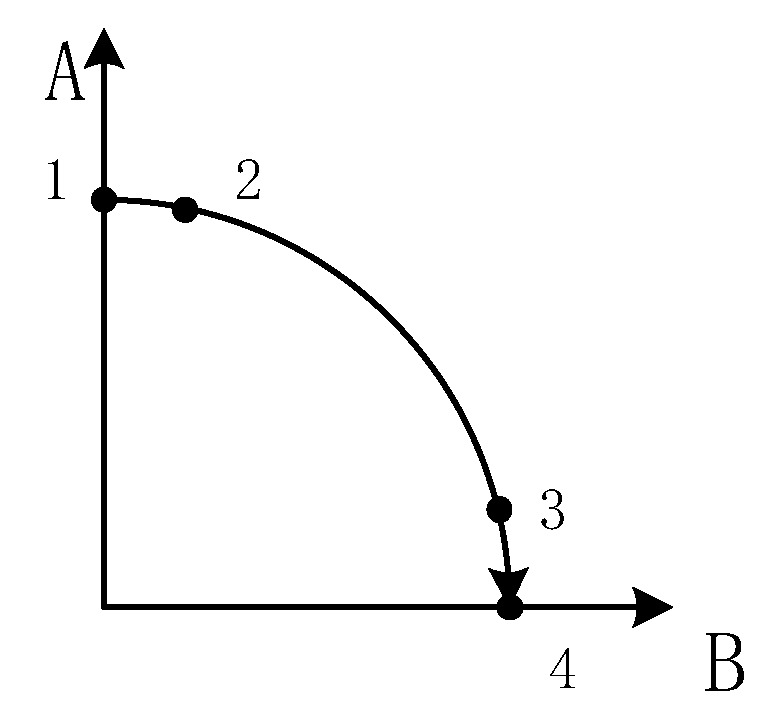
Rotation process.

**Figure 6:**
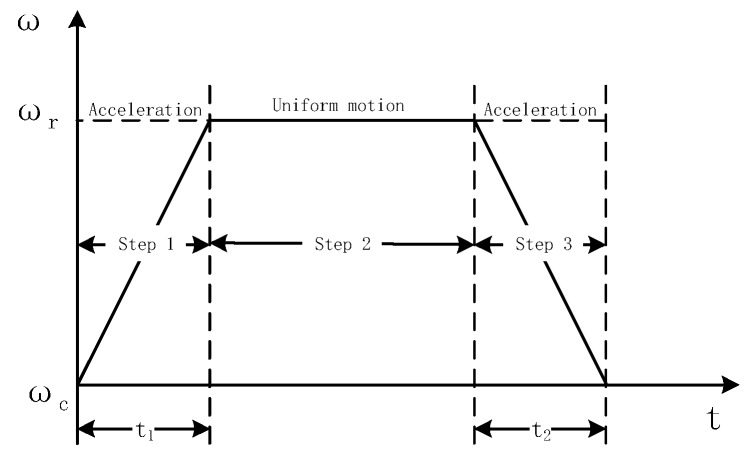
Diagram of the rotating angular velocity.

Ideally, the rotation process should be at a constant speed. However, in practical applications, the process of changing the rotational mechanism from one angular rate to another angular rate inevitably undergoes angular acceleration and angular deceleration. In order to facilitate analysis and calculation, here we consider the residual angular rate to be 0°. The rotation process is shown in Figure 5, and the change in the angular rate of rotation is shown in Figure 6.

The rotation mechanism is set from the residual angular rate to the modulation angular rate, that is, the 1 to 2 process shown in Figure 6 is an angular acceleration process. We assume that with 0° as the initial angle, the motion model of the process is established as

(6) $$\theta_{1}=\frac{1}{2}\chi \cdot t_{1}^{2}$$

where $\theta_{1}$ represents the angle at which the angular acceleration process is rotated, χ is the angular acceleration, $t_{1}$ represents the angular acceleration time, $\omega_{r}$ represents the angular velocity at the final moment of the acceleration motion, and $\omega_{r}=\chi t$. The motion model of the deceleration process is similar to the acceleration process and will not be described here.

Combined with the IMU constant drift modulation form in the modulation process formula of Equation (2), the output of the IMU constant drift during the angular acceleration motion of the rotating mechanism is obtained under the navigation coordinate system:

(7) $$\left[\begin{array}{c} \varepsilon_{E}\\ \varepsilon_{N}\\ \varepsilon_{U} \end{array}\right]=C_{b}^{n}C_{s}^{b}\varepsilon^{s}=\left[\begin{array}{c} \varepsilon_{x}^{s}\\ \varepsilon_{y}^{s}\cos\left(\frac{1}{2}\chi \cdot t_{1}^{2}\right)-\varepsilon_{z}^{s}\sin\left(\frac{1}{2}\chi \cdot t_{1}^{2}\right)\\ \varepsilon_{y}^{s}\sin\left(\frac{1}{2}\chi \cdot t_{1}^{2}\right)+\varepsilon_{z}^{s}\cos\left(\frac{1}{2}\chi \cdot t_{1}^{2}\right) \end{array}\right]$$

Then the cumulative result of the sensor constant drift during the $t_{1}$ period is:

(8) $$\int_{0}^{t_{1}}C_{b}^{n}C_{s}^{b}\varepsilon^{s}dt=\left[\begin{array}{c} \varepsilon_{x}^{s}t_{1}\\ \varepsilon_{y}^{s}\int_{0}^{t_{1}}\cos\left(\frac{1}{2}\chi \cdot t_{1}^{2}\right)dt_{1}-\varepsilon_{z}^{s}\int_{0}^{t_{1}}\sin\left(\frac{1}{2}\chi \cdot t_{1}^{2}\right)dt_{1}\\ \varepsilon_{y}^{s}\int_{0}^{t_{1}}\sin\left(\frac{1}{2}\chi \cdot t_{1}^{2}\right)dt_{1}+\varepsilon_{z}^{s}\int_{0}^{t_{1}}\cos\left(\frac{1}{2}\chi \cdot t_{1}^{2}\right)dt_{1} \end{array}\right]$$

Since $\sin\frac{1}{2}\chi \cdot t_{1}^{2}$ and $\cos\frac{1}{2}\chi \cdot t_{1}^{2}$ cannot be directly integrated, the Taylor expansion simplification integral process is used here. We reserve the first two terms of Taylor’s expansion and obtain the integral:

(9) $$\left\{ \begin{array}{l} \int_{0}^{t_{1}}\sin\left(\frac{1}{2}\chi \cdot t_{1}^{2}\right)dt_{1}\approx \frac{\chi \cdot t_{1}^{3}}{6}-\frac{\chi^{3}\cdot t_{1}^{7}}{336}\\ \int_{0}^{t_{1}}\cos\left(\frac{1}{2}\chi \cdot t_{1}^{2}\right)dt_{1}\approx t_{1}-\frac{\chi^{2}\cdot t_{1}^{5}}{40} \end{array}\right.$$

Substituting Equation (9) into (8) gives:

(10) $$\int_{0}^{t_{1}}C_{b}^{n}C_{s}^{b}\varepsilon^{s}dt=\left[\begin{array}{c} \varepsilon_{x}^{s}t_{1}\\ \varepsilon_{y}^{s}\left(t_{1}-\frac{\chi^{2}\cdot t_{1}^{5}}{40}\right)-\varepsilon_{z}^{s}\left(\frac{\chi \cdot t_{1}^{3}}{6}-\frac{\chi^{3}\cdot t_{1}^{7}}{336}\right)\\ \varepsilon_{z}^{s}\left(t_{1}-\frac{\chi^{2}\cdot t_{1}^{5}}{40}\right)+\varepsilon_{y}^{s}\left(\frac{\chi \cdot t_{1}^{3}}{6}-\frac{\chi^{3}\cdot t_{1}^{7}}{336}\right) \end{array}\right]$$

Equation (10) is the cumulative result of the IMU constant drift along the navigation system during the angular acceleration of the rotating mechanism. Since the IMU rotates around the *x*-axis, the sensor error in the direction of the rotation axis is not affected by the angular shifting motion.

We assume that the three-axis MEMS gyro constant error is 5°/h, the angular acceleration of the rotating mechanism is 3°/s, and the angular acceleration time is 2 s. When substituting the above set values into Equation (10), it can be found through the calculation that the cumulative output errors of $\varepsilon_{N}$ and $\varepsilon_{U}$ in this period are 9.3474 × 10^−6^° and 1.0024 × 10^−5^°. Assuming that there are multiple angular acceleration motions for one error modulation process, the error will increase as the navigation time increases; this has a bad effect on the effects of rotational modulation.

It can be seen from the calculation that in the same angle range, the accumulated error of the IMU along the navigation system in the acceleration process of 1 to 2 is the same as the deceleration process of 2 to 1, and that the analysis method is the same as the above process, so it will not be described here. From this, we conclude that the conclusions obtained by analyzing the angular acceleration of the rotating mechanism apply to the angular deceleration motion.

### 3.2. Modeling and Analysis of Rotation Rate Instability Error

Since the motor needs to follow the movement of the missile in real time, it is inevitable to generate the fluctuation and overshoot of the rotation angular rate. It is assumed that the motor output speed instability error during the acceleration-deceleration process is a dynamic error $\delta \omega_{1}$, and that the speed instability error in the uniform speed process is a steady state error $\delta \omega_{2}$. Furthermore, the dynamic error is greater than the steady state error. $C_{s_{1}}^{b}$ and $C_{s_{2}}^{b}$ are the direction cosine matrix between the S-frame and the B-frame in the acceleration-deceleration and uniform speed rotation process, respectively, and can be described as (11):

(11) $$C_{s_{1}}^{b}=\left[\begin{array}{ccc} 1 & 0 & 0\\ 0 & \cos\left(\frac{1}{2}\chi t+\delta \omega_{1}\right)t_{1} & -\sin\left(\frac{1}{2}\chi t+\delta \omega_{1}\right)t_{1}\\ 0 & \sin\left(\frac{1}{2}\chi t+\delta \omega_{1}\right)t_{1} & \cos\left(\frac{1}{2}\chi t+\delta \omega_{1}\right)t_{1} \end{array}\right]\begin{array}{c} , \end{array}C_{s_{2}}^{b}=\left[\begin{array}{ccc} 1 & 0 & 0\\ 0 & \cos\left(\omega +\delta \omega_{2}\right)t_{2} & -\sin\left(\omega +\delta \omega_{2}\right)t_{2}\\ 0 & \sin\left(\omega +\delta \omega_{2}\right)t_{2} & \cos\left(\omega +\delta \omega_{2}\right)t_{2} \end{array}\right]$$

Here, it is assumed that the time of the acceleration process and deceleration process is equal to $t_{1}$ and that the time of uniform velocity is $t_{2}$. The previous RSINS adopts a reciprocating rotation scheme, and when $\delta \omega_{2}t_{2}$ and $\frac{1}{2}\chi t_{1}^{2}$ are small angles, the attitude errors caused by the rotation rate instability error in a half rotation cycle (one counterclockwise cycle) can be described by Equation (12).

(12) $$\psi_{D}=2\int_{0}^{t_{1}}C_{s_{2}}^{b}\varepsilon^{s}dt+\int_{0}^{t_{2}}C_{s_{2}}^{b}\varepsilon^{s}dt\approx \left[\begin{array}{c} \varepsilon_{x}^{s}\left(t_{2}+2t_{1}\right)\\ \varepsilon_{y}^{s}\left(\frac{\sin\left(\theta_{1}+\delta \omega_{2}t_{2}\right)-\sin\theta_{1}}{\omega }+2t_{1}\right)+\varepsilon_{z}^{s}\left(\frac{\cos\left(\theta_{1}+\delta \omega_{2}t_{2}\right)-\cos\theta_{1}}{\omega }-\frac{\chi t_{1}^{3}}{3}\right)\\ -\varepsilon_{y}^{s}\left(\frac{\cos\left(\theta_{1}+\delta \omega_{2}t_{2}\right)-\cos\theta_{1}}{\omega }-\frac{\chi t_{1}^{3}}{3}\right)+\varepsilon_{z}^{s}\left(\frac{\sin\left(\theta_{1}+\delta \omega_{2}t_{2}\right)-\sin\theta_{1}}{\omega }+2t_{1}\right) \end{array}\right]$$

According to Equation (12), we know that the attitude errors caused by the rotation rate instability error perpendicular to the rotation axis cannot be completely offset and that the north and south attitude errors accumulate over time.

## 4. Analysis and Implementation of a New Rotation Scheme

In the SSINS, a good rotation scheme should coincide with the actual error’s own character. After the key errors of the SSINS are determined, the rotation scheme should be designed with an aim to offsetting these.

The above analysis of the angular rate of the motor’s actual output shows that the acceleration-deceleration process and the instability of the angular rate seriously affect the rotary modulation effect so as to affect the accuracy of the navigation solution. Hence, the design of the rotation scheme in the high-rotation environment should be combined with the motor’s actual output to design a reasonable solution that can suppress the sensor error to the greatest extent.

With reference to the motor actual output modulation angular rate model in the previous section and by analyzing the existing rotation scheme used in the missile-borne environment, a new rotation modulation scheme is inspired and proposed.

### 4.1. Inspiration and Design of the New Rotation Scheme

Constrained by factors such as small space and low cost in the missile-borne application environment, the system is not suitable for multi-axis rotation schemes. The system adopts a single-axis rotary modulation scheme and selects the *X*-axis as the modulation axis. The motor thus realizes the dual functions of rotation reduction and rotation modulation and the cost is reduced.

**Figure 7:**
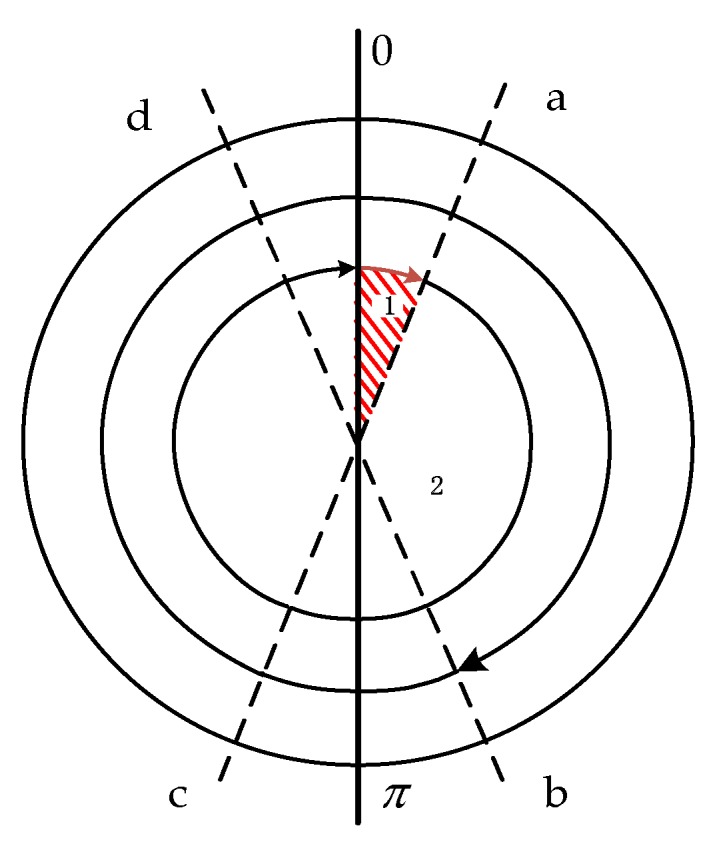
Single axis continuous rotation.

**Figure 8:**
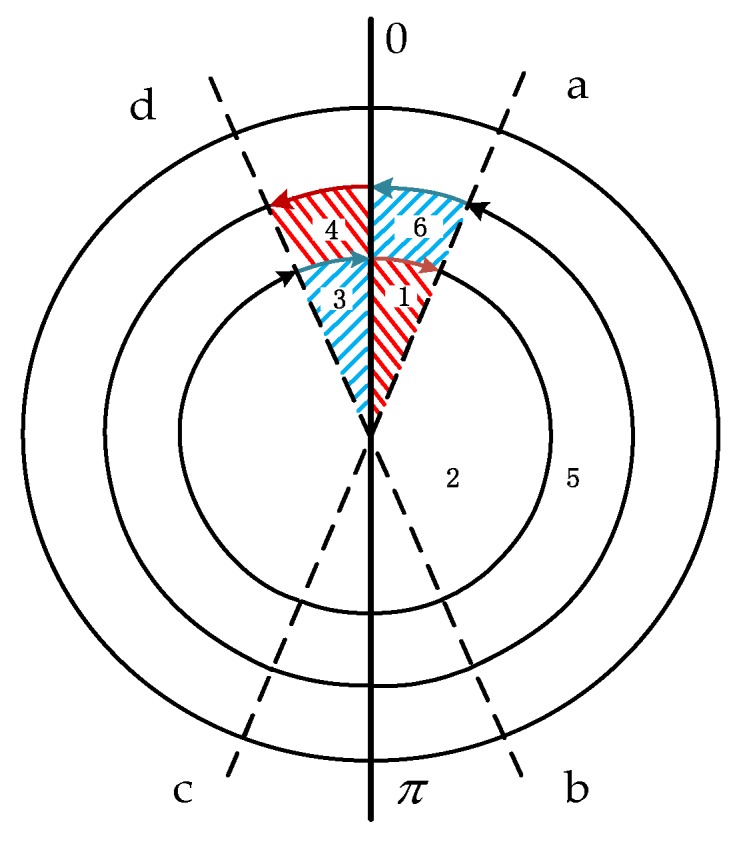
Single reciprocating rotation.

The following are two existing rotation schemes that take into account the actual output of the motor. Figure 7 shows a single-axis continuous rotation and Figure 8 shows a single-axis reciprocating rotation. In Figure 7, Process 1 represents the acceleration stage of the motor start and Process 2 represents the continuous reciprocating rotation. In Figure 8, Process 1 indicates a positive acceleration stage, Process 2 is a constant speed rotation, Process 3 is a deceleration stage in which the motor is stopped, and Processes 4, 5, and 6 indicate a reverse rotation process.

The essence of rotational modulation is that the inertial sensor has a symmetrical motion state at a symmetrical position to achieve self-compensation of the error. The symmetry of time, space, and motion state is the key to achieving complete modulation of error. The unidirectional continuous rotation motivates significant navigation error if the scale factor in the rotation axis cannot be completely removed [29]. Therefore, taking the conventional reciprocating rotation shown in Figure 8 as an example, the modulation effect of the rotation scheme is analyzed. The 0-a and d-0 processes are the angular variable motion of the rotating mechanism, and, at the same time, the angular rate overshoot phenomenon is inevitable at the starting time, and the corresponding π-c and b-π processes are uniform speed stages. Obviously, the rotational symmetry is destroyed, resulting in a sensor error that cannot be completely suppressed or even caused by larger errors.

In work [30], the influence of angular variable motion on the rotating strapdown inertial navigation system is analyzed. On this basis, [31] designed 8 times 4-position stop-and-go rotation schemes to offset the effects of acceleration and deceleration on device errors, and the effect was good. However, this rotation scheme requires high precision in motor control. If the rotation process of the motor cannot be accurately controlled, it may be counterproductive.

**Figure 9:**
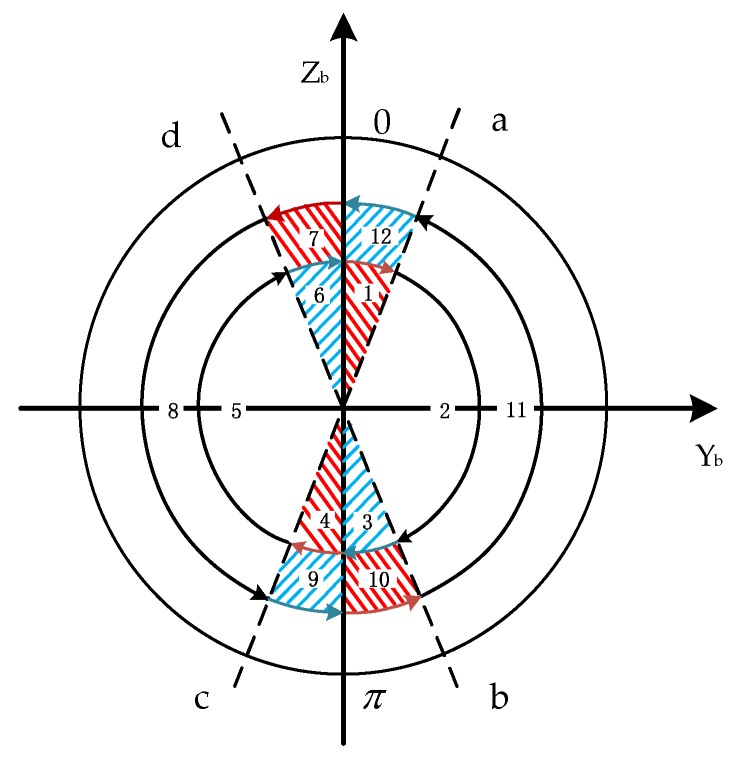
Diagram of the rotation sequence.

As can be seen from [30,31], the sensor error can be suppressed by changing the rotation scheme. Inspired by the ‘symmetry rotation criterion’, we propose a 2-position continuous reciprocating rotation scheme as shown in Figure 9. The new rotation scheme not only can make the 0-a phase and the corresponding π-c phase the acceleration process, but also make the d-0 phase and the corresponding b-π phase the same as the deceleration process. This realizes the symmetry of rotation, suppresses the propagation of device errors, and does not require the motor to have high control precision, meaning it is suitable for application in a missile-borne environment.

Many previous works have considered the change in angular rate as a process of saltation when designing a rotation scheme. Conversely, the new rotation scheme sets the acceleration and deceleration process and takes into account the error characteristics of the actual motor output in a high-rotation environment.

As shown in Figure 9, in the new scheme, a complete reciprocating rotation cycle includes a rotation cycle (360 degrees) about the *X*-axis in the clockwise (positive) direction and then a rotation cycle about the *X*-axis in the counter-clockwise (negative) direction, with its time being T. The demarcation lines between the acceleration-deceleration process and the uniform rotation are a, b, c, and d, respectively, and the red area in the Figure 9 represents the acceleration process while the blue area represents the deceleration process. The rotation scheme is designed in this way because the acceleration process and deceleration process of the new scheme is symmetrical with respect to the entire rotation period, meaning that we can use the idea that the same state of the symmetric positions can cancel each other, and therefore, the maximum degree of suppression of the device error is achieved in the case where there is an error in the motor output modulation angular rate. The specific expression of the rotation process is as shown in Equation (13). The rotation sequence diagram is shown in Figure 9.

(13) $$\{\begin{array}{llllll} Process\;1 & & \theta_{1}=\frac{1}{2}\chi \cdot t_{1}^{2} & & 0<t_{1}\leq t_{a} & \\ Process\;2 & & \theta_{2}=\omega \cdot t_{2} & & t_{a}<t_{2}\leq t_{b} & \\ Process\;3 & & \theta_{3}=\pi -\frac{1}{2}\chi \cdot t_{3}^{2} & & t_{b}<t_{3}\leq \pi & \\ Process\;4 & & \theta_{4}=\pi +\frac{1}{2}\chi \cdot t_{4}^{2} & & \pi <t_{4}\leq t_{c} & \\ Process\;5 & & \theta_{5}=\omega \cdot t_{5} & & t_{c}<t_{5}\leq t_{d} & \\ Process\;6 & & \theta_{6}=2\pi -\frac{1}{2}\chi \cdot t_{6}^{2} & & t_{d}<t_{6}\leq 2\pi & \end{array}$$

In order to simplify the analysis, here we assume that the value of the acceleration χ during the acceleration and deceleration process are the same and furthermore $t_{1}=t_{3}=t_{4}=t_{6}=t_{7}=t_{9}=t_{10}=t_{12}$, meaning $\theta_{1}=\theta_{3}=\theta_{4}=\theta_{6}=\theta_{7}=\theta_{9}=\theta_{10}=\theta_{12}$ and $\theta_{2}=\theta_{5}=\theta_{8}=\theta_{11}$. 7, 8, 9, 10, 11, and 12 are a counter-clockwise rotation process; the motion of 1, 2, 3, 4, 5, and 6 is also a clockwise rotation process, but in the opposite direction.

Starting from the modulation angular rate error model of the motor’s actual output in the previous section, the suppression effect of the new rotation scheme on the residual error of the acceleration and deceleration process and the suppression effect on the modulation angular rate instability error are analyzed.

### 4.2. Residual Error Suppression During Acceleration-Deceleration Process

#### 4.2.1. Constant Drift

When the IMU is rotating in the clockwise direction, Process 1 as shown in Figure 9 is an acceleration process, and the modulation form of the gyro’s constant drift is:

(14) $$\delta \omega_{1}^{n}=C_{b}^{n}C_{s}^{b}\varepsilon^{s}=\left[\begin{array}{ccc} 1 & 0 & 0\\ 0 & \cos\theta_{1} & \sin\theta_{1}\\ 0 & -\sin\theta_{1} & \cos\theta_{1} \end{array}\right]\left[\begin{array}{c} \varepsilon_{x}^{s}\\ \varepsilon_{y}^{s}\\ \varepsilon_{z}^{s} \end{array}\right]=\left[\begin{array}{c} \varepsilon_{x}^{s}\\ \varepsilon_{y}^{s}\cos\theta_{1}+\varepsilon_{z}^{s}\sin\theta_{1}\\ -\varepsilon_{y}^{s}\sin\theta_{1}+\varepsilon_{z}^{s}\cos\theta_{1} \end{array}\right]$$

(15) $$\delta \psi_{1}=\int_{0}^{t_{a}}C_{b}^{n}C_{s}^{b}\varepsilon^{s}dt=\left[\begin{array}{c} \varepsilon_{x}^{s}t_{1}\\ \varepsilon_{y}^{s}\int_{0}^{t_{a}}\cos\left(\frac{1}{2}\chi \cdot t_{1}^{2}\right)dt_{1}+\varepsilon_{z}^{s}\int_{0}^{t_{a}}\sin\left(\frac{1}{2}\chi \cdot t_{1}^{2}\right)dt_{1}\\ -\varepsilon_{y}^{s}\int_{0}^{t_{a}}\sin\left(\frac{1}{2}\chi \cdot t_{1}^{2}\right)dt_{1}+\varepsilon_{z}^{s}\int_{0}^{t_{a}}\cos\left(\frac{1}{2}\chi \cdot t_{1}^{2}\right)dt_{1} \end{array}\right]$$

In order to facilitate calculations, here we take the first item approximation of *A* and *B* by the Taylor formula:

(16) $$\left\{ \begin{array}{l} A=\int_{0}^{t_{a}}\sin\left(\frac{1}{2}\chi \cdot t_{1}^{2}\right)dt_{1}\approx \frac{\chi \cdot t_{1}^{3}}{6}\\ B=\int_{0}^{t_{a}}\cos\left(\frac{1}{2}\chi \cdot t_{1}^{2}\right)dt_{1}\approx t_{1} \end{array}\right.$$

So, according to Equation (16), the attitude errors, during the acceleration phase, can be expressed as follows:

(17) $$\delta \psi_{1}=\left[\begin{array}{c} \varepsilon_{x}^{s}t_{1}\\ \varepsilon_{y}^{s}t_{1}+\varepsilon_{z}^{s}\frac{\chi t_{1}^{3}}{6}\\ -\varepsilon_{z}^{s}t_{1}+\varepsilon_{y}^{s}\frac{\chi t_{1}^{3}}{6} \end{array}\right]$$

As shown in Process 4 of Figure 9, the IMU performs the same-angle acceleration motion with π as the initial position. In this process, the integration result of the gyro output error along the navigation coordinate system is:

(18) $$\begin{array}{l} \delta \psi_{4}=\int_{\pi }^{t_{c}}C_{b}^{n}C_{s}^{b}\varepsilon^{s}dt=\left[\begin{array}{c} \varepsilon_{x}^{s}t_{1}\\ \varepsilon_{y}^{s}\int_{0}^{t_{1}}\cos\left(\pi +\frac{1}{2}\chi \cdot t_{1}^{2}\right)dt_{1}+\varepsilon_{z}^{s}\int_{0}^{t_{1}}\sin\left(\pi +\frac{1}{2}\chi \cdot t_{1}^{2}\right)dt_{1}\\ -\varepsilon_{y}^{s}\int_{0}^{t_{1}}\sin\left(\pi +\frac{1}{2}\chi \cdot t_{1}^{2}\right)dt_{1}+\varepsilon_{z}^{s}\int_{0}^{t_{1}}\cos\left(\pi +\frac{1}{2}\chi \cdot t_{1}^{2}\right)dt_{1} \end{array}\right]\\ =\left[\begin{array}{c} \varepsilon_{x}^{s}t_{1}\\ -\varepsilon_{y}^{s}\int_{0}^{t_{1}}\cos\left(\frac{1}{2}\chi \cdot t_{1}^{2}\right)dt_{1}-\varepsilon_{z}^{s}\int_{0}^{t_{1}}\sin\left(\frac{1}{2}\chi \cdot t_{1}^{2}\right)dt_{1}\\ +\varepsilon_{y}^{s}\int_{0}^{t_{1}}\sin\left(\frac{1}{2}\chi \cdot t_{1}^{2}\right)dt_{1}-\varepsilon_{z}^{s}\int_{0}^{t_{1}}\cos\left(\frac{1}{2}\chi \cdot t_{1}^{2}\right)dt_{1} \end{array}\right]=\left[\begin{array}{c} \varepsilon_{x}^{s}t_{1}\\ -\varepsilon_{y}^{s}t_{1}-\varepsilon_{z}^{s}\frac{\chi t_{1}^{3}}{6}\\ +\varepsilon_{z}^{s}t_{1}-\varepsilon_{y}^{s}\frac{\chi t_{1}^{3}}{6} \end{array}\right] \end{array}$$

Through this calculation, we can find the law. In Process 1 and Process 4, the gyro drifts in the direction which is perpendicular to the rotation axis have a similar form, but their sign is the opposite. Hence, the acceleration movement in the same direction of the symmetrical position can eliminate the accumulation of errors caused by the angular acceleration movement of the rotating mechanism. Adding Equations (17) and (18) gives the following result:

(19) $$\delta \psi_{1}+\delta \psi_{4}=\left[\begin{array}{c} 2\varepsilon_{x}^{s}t_{1}\\ 0\\ 0 \end{array}\right]$$

Process 3 and Process 6 are deceleration processes, which are obtained by the same calculation process as the acceleration process. The integration results of the two deceleration processes are added as follows:

(20) $$\delta \psi_{3}+\delta \psi_{6}=\left[\begin{array}{c} 2\varepsilon_{x}^{s}t_{1}\\ 0\\ 0 \end{array}\right]$$

As shown in Figure 9, Process 2 and Process 5 are uniform motion processes, and the integration results are added as follows:

(21) $$\delta \psi_{2}=\int_{t_{a}}^{t_{b}}C_{b}^{n}C_{s}^{b}\varepsilon^{s}=\left[\begin{array}{c} \varepsilon_{x}^{s}t_{2}\\ \varepsilon_{y}^{s}\frac{\sin\left(\theta_{1}+\omega t_{2}\right)-\sin\theta_{1}}{\omega }+\varepsilon_{z}^{s}\frac{\cos\left(\theta_{1}+\omega t_{2}\right)-\cos\theta_{1}}{\omega }\\ -\varepsilon_{y}^{s}\frac{\cos\left(\theta_{1}+\omega t_{2}\right)-\cos\theta_{1}}{\omega }+\varepsilon_{z}^{s}\frac{\sin\left(\theta_{1}+\omega t_{2}\right)-\sin\theta_{1}}{\omega } \end{array}\right]$$

(22) $$\delta \psi_{5}=\int_{t_{c}}^{t_{d}}C_{b}^{n}C_{s}^{b}\varepsilon^{s}=\left[\begin{array}{c} \varepsilon_{x}^{s}t_{2}\\ -\varepsilon_{y}^{s}\frac{\sin\left(\theta_{1}+\omega t_{2}\right)-\sin\theta_{1}}{\omega }-\varepsilon_{z}^{s}\frac{\cos\left(\theta_{1}+\omega t_{2}\right)-\cos\theta_{1}}{\omega }\\ +\varepsilon_{y}^{s}\frac{\cos\left(\theta_{1}+\omega t_{2}\right)-\cos\theta_{1}}{\omega }-\varepsilon_{z}^{s}\frac{\sin\left(\theta_{1}+\omega t_{2}\right)-\sin\theta_{1}}{\omega } \end{array}\right]$$

(23) $$\delta \psi_{2}+\delta \psi_{5}=\left[\begin{array}{c} 2\varepsilon_{x}^{s}t_{2}\\ 0\\ 0 \end{array}\right]$$

The integration result of the gyro output error in the clockwise direction along the navigation coordinate system is:

(24) $$\delta \psi_{clockwise}=\left[\begin{array}{c} 4\varepsilon_{x}^{s}t_{1}+2\varepsilon_{x}^{s}t_{2}\\ 0\\ 0 \end{array}\right]$$

The counterclockwise motion of the IMU mounted on the rotating mechanism is the same as the clockwise motion, so the gyro output error in the counterclockwise motion is calculated as above. The attitude errors caused by the gyro drifts in the counterclockwise direction are added as Equation (25) and the attitude errors caused by the gyro drifts in a complete cycle (one clockwise cycle plus one counterclockwise cycle) can be described by Equation (26):

(25) $$\delta \psi_{counterclockwise}=\left[\begin{array}{c} 4\varepsilon_{x}^{s}t_{1}+2\varepsilon_{x}^{s}t_{2}\\ 0\\ 0 \end{array}\right]$$

(26) $$\delta \psi_{clockwise}+\delta \psi_{counterclockwise}=\left[\begin{array}{c} 8\varepsilon_{x}^{s}t_{1}+4\varepsilon_{x}^{s}t_{2}\\ 0\\ 0 \end{array}\right]=\left[\begin{array}{c} \varepsilon_{x}^{s}T\\ 0\\ 0 \end{array}\right]$$

As can be seen from the above, differently from the traditional single-axis continuous rotation scheme and reciprocating rotation scheme, the new rotation scheme effectively eliminates the influence of the motor acceleration-deceleration process on the rotation modulation effect. Therefore, the gyro drift is completely eliminated in the direction perpendicular to the rotation axis and does not cause an accumulation of the missile attitude angle error; the drift on the rotating shaft propagates according to the original law.

The optimal design of the rotation scheme needs to be considered for many factors, and, moreover, there are always a few drawbacks in the real system. After the key errors are confirmed, the rotation scheme should be designed with an aim to compensate for these. In the missile-borne rotation semi-strapdown inertial navigation system (RSSINS), the sensor we use is the MEMS inertial sensor, and the magnitude of the constant drift error is much larger than the scale factor error and the installation error. Hence, the purposes for using the rotation modulation technique are to decrease or not induce the influence of the inertia sensor scale factor error and the installation error on the navigation accuracy, and, most importantly, to completely eliminate the influence of the IMU constant drift.

#### 4.2.2. Scale Factors

The scale factor is obtained in calibration testing and stored in the navigation computer in advance. The measured value is multiplied by the scale factor to obtain the actual specific force or angular velocity, which is used to calculate the IMU measurement output information. However, due to the test environment in the calibration test process being quite different from the actual application environment, the scale factor of the inertial sensor in the actual navigation work is different from the scale factor pre-stored to the computer.

Then, the IMU rotates with respect to the *X*-axis. The gyro outputs in the S-frame are shown as Equation (27), and the gyro output errors caused by scale factors can be described by Equation (28). When the IMU moves at a constant speed, A=ω, B=ωt, and when the IMU performs the acceleration-deceleration motion process, A=χt, B=12χt2.

(27) $$\left[\begin{array}{c} \omega_{isx}^{s}\\ \omega_{isy}^{s}\\ \omega_{isz}^{s} \end{array}\right]=C_{b}^{s}\omega_{is}^{b}=\left[\begin{array}{ccc} 1 & 0 & 0\\ 0 & \cos B & \sin B\\ 0 & -\sin B & \cos B \end{array}\right]\left[\begin{array}{c} A\\ \omega_{ie}\cos L\\ \omega_{ie}\sin L \end{array}\right]=\left[\begin{array}{c} A\\ \omega_{ie}\cos L\cos B+\omega_{ie}\sin L\sin B\\ -\omega_{ie}\cos L\sin B+\omega_{ie}\sin L\cos B \end{array}\right]$$

(28) $$\begin{array}{l} \delta \omega_{Kg}^{n}=C_{b}^{n}C_{s}^{b}\delta \omega_{Kg}^{s}=\left[\begin{array}{ccc} 1 & 0 & 0\\ 0 & 1 & 0\\ 0 & 0 & 1 \end{array}\right]\left[\begin{array}{ccc} 1 & 0 & 0\\ 0 & \cos B & \sin B\\ 0 & -\sin B & \cos B \end{array}\right]\left[\begin{array}{ccc} K_{gx} & 0 & 0\\ 0 & K_{gy} & 0\\ 0 & 0 & K_{gz} \end{array}\right]\omega_{is}^{s} \end{array}$$

where $\omega_{ie}$ denotes the earth rotation rate, $\omega_{is}^{s}$ is the ideal gyro output in the S-frame, L is the latitude, $\delta \omega_{is}^{s}$ is the sensor error caused by the scale factors, and $K_{gx}$, $K_{gy}$, and $K_{gz}$ are the scale factors for gyros along the X, Y, and Z axes, respectively. Through calculation we can obtain the attitude errors of the gyro caused by the scale factors in a complete cycle (one clockwise cycle plus one counterclockwise cycle), which can be described by Equation (29):

(29) $$\delta \psi_{K_{g}}=\int_{0}^{T}C_{b}^{n}C_{s}^{b}\delta \omega_{K_{g}}^{s}=\left[\begin{array}{c} \begin{array}{l} 0 \end{array}\\ 2\omega_{ie}\cos L\left(K_{gy}-K_{gz}\right)t_{2}+\frac{2}{\pi }\omega_{ie}\cos L\left(K_{gy}+K_{gz}\right)\left(4\theta_{1}-\sin2\theta_{1}\right)t_{1}\\ -2\omega_{ie}\sin L\left(K_{gy}-K_{gz}\right)t_{2}+\frac{2}{\pi }\omega_{ie}\sin L\left(K_{gy}+K_{gz}\right)\left(4\theta_{1}-\sin2\theta_{1}\right)t_{1} \end{array}\right]$$

It can be seen from Equation (29) that the acceleration-deceleration process has no influence on the eastward attitude, but attitude errors in the north and up directions accumulate over time. Compared to the single-axis continuous rotation scheme, the coupling error term between the gyro scale factor and the rotational angular velocity do not appear in the direction of the rotation axis of this scheme. The coupling error term is equivalent to the gyro drift in the direction of the rotating axis, which causes a large position error of the inertial navigation system. Therefore, this scheme can effectively ensure the accuracy of the eastward position. Compared with traditional single-axis continuous positive and negative rotation, this rotation scheme takes into account the acceleration-deceleration process of the indexing mechanism. Considering the limit, that is, when $\theta_{1}=0$, $t_{1}=0$, this formula is equal to the attitude error caused by the scale factor of the ideal forward and reverse rotation scheme, and the attitude error is theoretically minimized at this time. However, the actual output angular rate of the motor must undergo acceleration and deceleration, and the attitude error caused by the scale factor is related to $\theta_{1}$ and $t_{1}$.

#### 4.2.3. Installation Errors

For the IMU, the three gyros should be mounted perpendicular to each other in accordance with the three axes of the carrier coordinate system. However, in the actual application, although it is compensated for, there are still installation errors, and this will bring errors to the calculation of the attitude. Similarly to the calculation method of the scale factor error, the gyro outputs in the S-frame are shown as Equation (30), and the gyro outputs errors caused by installation errors can be described by Equation (31).

(30) $$\left[\begin{array}{c} \omega_{isx}^{s}\\ \omega_{isy}^{s}\\ \omega_{isz}^{s} \end{array}\right]=C_{b}^{s}\omega_{is}^{b}=\left[\begin{array}{ccc} 1 & 0 & 0\\ 0 & \cos B & \sin B\\ 0 & -\sin B & \cos B \end{array}\right]\left[\begin{array}{c} A\\ \omega_{ie}\cos L\\ \omega_{ie}\sin L \end{array}\right]=\left[\begin{array}{c} A\\ \omega_{ie}\cos L\cos B+\omega_{ie}\sin L\sin B\\ -\omega_{ie}\cos L\sin B+\omega_{ie}\sin L\cos B \end{array}\right]$$

(31) $$\begin{array}{l} \delta \omega_{Eg}^{n}=C_{b}^{n}C_{s}^{b}\delta \omega_{Kg}^{s}=\left[\begin{array}{ccc} 1 & 0 & 0\\ 0 & 1 & 0\\ 0 & 0 & 1 \end{array}\right]\left[\begin{array}{ccc} 1 & 0 & 0\\ 0 & \cos B & \sin B\\ 0 & -\sin B & \cos B \end{array}\right]\left[\begin{array}{ccc} 0 & K_{gxy} & K_{gxz}\\ K_{gyx} & 0 & K_{gyz}\\ K_{gzx} & K_{gzy} & 0 \end{array}\right]\omega_{is}^{s} \end{array}$$

Here, $\delta \omega_{E_{g}}^{s}$ represents the gyro errors caused by the installation errors and $K_{gij}$ represents the installation error parameter between the *i* axis and the *j* axis (*i*, *j* = *X*, *Y*, *Z*). When the system adopts a new rotation scheme, the attitude errors for a complete cycle can be described by:

(32) $$\delta \psi_{k_{g}}=\int_{0}^{T}C_{b}^{n}C_{s}^{b}\delta \omega_{k_{g}}^{s}=\left[\begin{array}{c} \frac{\omega_{ie}\left(K_{gxy}\cos L+K_{gxz}\sin L\right)}{\pi }\cdot 4t_{2}+\frac{\omega_{ie}\left(2\theta_{1}-\sin\theta_{1}\right)}{\pi }\cdot 8t_{1}\\ 2\omega_{ie}\sin L\left(K_{gyz}+K_{gzy}\right)t_{2}+\frac{\omega_{ie}\sin L\left(K_{gyz}-K_{gzy}\right)\left(2\theta_{1}-\sin\theta_{1}\right)}{\pi }\cdot 4t_{1}\\ 2\omega_{ie}\cos L\left(K_{gyz}+K_{gzy}\right)t_{2}-\frac{\omega_{ie}\cos L\left(K_{gyz}-K_{gzy}\right)\left(2\theta_{1}-\sin\theta_{1}\right)}{\pi }\cdot 4t_{1} \end{array}\right]$$

It can be seen from Equation (32) that the acceleration-deceleration process has an impact on the attitude precision in all three directions. Considering the limit of when $\theta_{1}=0$, $t_{1}=0$, this formula is equal to the attitude error caused by the installation errors of the ideal reciprocating rotation scheme, and the attitude error is theoretically minimized at this time. The attitude error caused by the scale factor is related to $\theta_{1}$ and $t_{1}$, not χ and $t_{1}$.

### 4.3. Suppression of Angular Rate Instability Error

Due to the RSSINS being applied in a high-rotation and high-dynamic environment, although the modulation angular rate of the new rotation scheme has no sharp change, the motor still needs to be adjusted according to the preset rotation scheme after completing the decrease in the rotation, so the motor’s angular rate instability error is inevitable. The modulation form of the gyro’s constant drift caused by the motor’s angular rate instability error in Process 1 can be described by Equation (33). Similarly, Process 4 can be expressed as Equation (34) and the Processes 2 and 5 can be expressed as Equations (35) and (36).

(33) $$\delta \omega_{1}^{n}=C_{b}^{n}C_{s}^{b}\varepsilon^{s}=\left[\begin{array}{ccc} 1 & 0 & 0\\ 0 & \cos\left(\frac{1}{2}\chi t+\delta \omega_{1}\right)t_{1} & \sin\left(\frac{1}{2}\chi t+\delta \omega_{1}\right)t_{1}\\ 0 & -\sin\left(\frac{1}{2}\chi t+\delta \omega_{1}\right)t_{1} & \cos\left(\frac{1}{2}\chi t+\delta \omega_{1}\right)t_{1} \end{array}\right]\left[\begin{array}{c} \varepsilon_{x}^{s}\\ \varepsilon_{y}^{s}\\ \varepsilon_{z}^{s} \end{array}\right]=\left[\begin{array}{c} \varepsilon_{x}^{s}\\ \varepsilon_{y}^{s}\cos\left(\frac{1}{2}\chi t+\delta \omega_{1}\right)t_{1}+\varepsilon_{z}^{s}\sin\left(\frac{1}{2}\chi t+\delta \omega_{1}\right)t_{1}\\ -\varepsilon_{y}^{s}\sin\left(\frac{1}{2}\chi t+\delta \omega_{1}\right)t_{1}+\varepsilon_{z}^{s}\cos\left(\frac{1}{2}\chi t+\delta \omega_{1}\right)t_{1} \end{array}\right]$$

(34) $$\delta \omega_{4}^{n}=C_{b}^{n}C_{s}^{b}\varepsilon^{s}=\left[\begin{array}{ccc} 1 & 0 & 0\\ 0 & \cos\left(\pi +\frac{1}{2}\chi t+\delta \omega_{1}\right)t_{1} & \sin\left(\pi +\frac{1}{2}\chi t+\delta \omega_{1}\right)t_{1}\\ 0 & -\sin\left(\pi +\frac{1}{2}\chi t+\delta \omega_{1}\right)t_{1} & \cos\left(\pi +\frac{1}{2}\chi t+\delta \omega_{1}\right)t_{1} \end{array}\right]\left[\begin{array}{c} \varepsilon_{x}^{s}\\ \varepsilon_{y}^{s}\\ \varepsilon_{z}^{s} \end{array}\right]=\left[\begin{array}{c} \varepsilon_{x}^{s}\\ -\varepsilon_{y}^{s}\sin\left(\frac{1}{2}\chi t+\delta \omega_{1}\right)t_{1}-\varepsilon_{z}^{s}\sin\left(\frac{1}{2}\chi t+\delta \omega_{1}\right)t_{1}\\ +\varepsilon_{y}^{s}\sin\left(\frac{1}{2}\chi t+\delta \omega_{1}\right)t_{1}{-}_{z}^{s}\cos\left(\frac{1}{2}\chi t+\delta \omega_{1}\right)t_{1} \end{array}\right]$$

(35) $$\delta \omega_{2}^{n}=C_{b}^{n}C_{s}^{b}\varepsilon^{s}=\left[\begin{array}{ccc} 1 & 0 & 0\\ 0 & \cos\left(\omega +\delta \omega_{2}\right)t_{2} & \sin\left(\omega +\delta \omega_{2}\right)t_{2}\\ 0 & -\sin\left(\omega +\delta \omega_{2}\right)t_{2} & \cos\left(\omega +\delta \omega_{2}\right)t_{2} \end{array}\right]\left[\begin{array}{c} \varepsilon_{x}^{s}\\ \varepsilon_{y}^{s}\\ \varepsilon_{z}^{s} \end{array}\right]=\left[\begin{array}{c} \varepsilon_{x}^{s}\\ \varepsilon_{y}^{s}\cos\left(\omega +\delta \omega_{2}\right)t_{2}+\varepsilon_{z}^{s}\sin\left(\omega +\delta \omega_{2}\right)t_{2}\\ -\varepsilon_{y}^{s}\sin\left(\omega +\delta \omega_{2}\right)t_{2}+\varepsilon_{z}^{s}\cos\left(\omega +\delta \omega_{2}\right)t_{2} \end{array}\right]$$

(36) $$\delta \omega_{2}^{n}=C_{b}^{n}C_{s}^{b}\varepsilon^{s}=\left[\begin{array}{ccc} 1 & 0 & 0\\ 0 & \cos\left(\pi +\omega +\delta \omega_{2}\right)t_{2} & \sin\left(\pi +\omega +\delta \omega_{2}\right)t_{2}\\ 0 & -\sin\left(\pi +\omega +\delta \omega_{2}\right)t_{2} & \cos\left(\pi +\omega +\delta \omega_{2}\right)t_{2} \end{array}\right]\left[\begin{array}{c} \varepsilon_{x}^{s}\\ \varepsilon_{y}^{s}\\ \varepsilon_{z}^{s} \end{array}\right]=\left[\begin{array}{c} \varepsilon_{x}^{s}\\ -\varepsilon_{y}^{s}\cos\left(\omega +\delta \omega_{2}\right)t_{2}-\varepsilon_{z}^{s}\sin\left(\omega +\delta \omega_{2}\right)t_{2}\\ +\varepsilon_{y}^{s}\sin\left(\omega +\delta \omega_{2}\right)t_{2}-\varepsilon_{z}^{s}\cos\left(\omega +\delta \omega_{2}\right)t_{2} \end{array}\right]$$

As can be seen from the above formula, the above modulation form of the gyro’s constant drift is caused by the motor’s speed output error, and in the symmetrical motion process, the form of modulation angular rate instability error has the same size and opposite direction. Therefore, the cumulative attitude errors caused by the motor’s modulation angular rate instability error in a complete cycle (one clockwise cycle plus one counterclockwise cycle) can be described by Equation (37):

(37) $$\delta \psi_{clockwise}+\delta \psi_{counterclockwise}=\left[\begin{array}{c} 8\varepsilon_{x}^{s}t_{1}+4\varepsilon_{x}^{s}t_{2}\\ 0\\ 0 \end{array}\right]=\left[\begin{array}{c} \varepsilon_{x}^{s}T\\ 0\\ 0 \end{array}\right]$$

It can be seen from Equation (37) that in the ideal case, the new rotation scheme can be completely modulated for the motor output error. In practical applications, due to the motor control characteristics, modulation angular rate instability errors are similar in form, so the new rotation scheme can offset most symmetrical angular rate output errors.

## 5. Discussion

The background to missile-borne applications is different from that of ship-borne applications. When the rotary modulation technique is applied in a ship-borne environment, due to its large internal space, a high-precision rotating mechanism can be used and the modulation angular rate setting is lower, making the modulation angular rate error smaller or even negligible. For the SSINS carried on conventional missiles, due to the limitations of cost and the particularity of the application background, the motor output angular rate inevitably produces large errors. The modulation angular rate error affects the navigation accuracy by affecting the error suppression effect of the rotational modulation. Therefore, it is necessary to design a rotation scheme that can suppress the device error propagation to the system to the maximum extent for the special application background of the missile.

As is well known, rotary modulation technology rotates the inertial sensor to perform periodic motion through the rotating mechanism, so as to complete the modulation of the inertial sensor constant error to achieve higher navigation accuracy. The constant errors of MEMS inertial sensors mainly include constant drift, scale factor error, and installation error. The constant drift of the MEMS inertial sensor is one to two orders of magnitude larger than the other two errors. Therefore, it is important to consider the error suppression effect for the constant drift. Due to the large error in the modulation angular in the missile-borne environment, an improper rotation scheme can greatly weaken the effect of modulation and even excite larger errors. Table 1 shows with or without motor output speed error, a comparison between the existing reciprocating rotation scheme and the new rotation scheme. As can be seen from Table 1, the new rotational modulation scheme can effectively suppress the modulation angular rate error. Furthermore, the new rotary modulation scheme utilizes the scheme itself, rather than other hardware aids or algorithms to compensate for errors in the modulation angular rate, thereby improving the accuracy of the navigation solution. In addition, this scheme uses fully the idea of rotational modulation in which the MIMU has the same movement process at the symmetrical position to offset the sensor errors.

In this paper, however, we simply modelled the modulation angular rate error model rather than exploring an accurate motor output modulation angular rate error model. If the motor rotation speed error is accurately modeled, it needs to be analyzed individually for different types of motors. According to the output characteristics of the motor, the motor rotation speed error has a certain symmetry at a symmetrical position, but it is not completely symmetrical. Therefore, the new rotation scheme can greatly suppress the sensor error in the missile-borne environment, but this error cannot be completely suppressed.

Furthermore, it is necessary to consider the relationship between the motor’s actual output angular rate and the preset modulation angular rate. The error propagation equation of the inertial sensor should also be comprehensively considered and the optimal angular rate and acceleration of the new rotation scheme further designed to achieve the optimal application of the new rotation scheme. Or, the modulation angular rate error of the motor output should be accurately modelled, with software or hardware-assisted methods used to accurately compensate while applying a new rotation scheme.

## 6. Testing Different Rotation Scheme on a Tri-Axial Rotation Table

**Figure 10:**
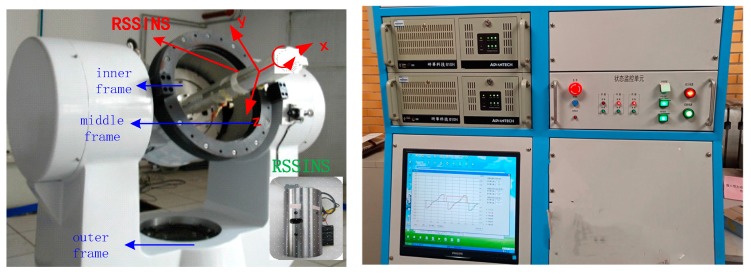
Rotating strapdown inertial navigation system (RSSINS) installation on tri-axial rotation table and a console.

In order to verify the practicality and effectiveness of the new rotation scheme in practical applications, the RSSINS was installed on a high-precision tri-axial flight simulator to carry out the experiment. The high-precision tri-axial flight simulator consisted of a tri-axial rotation table and a console as shown in Figure 10. The tri-axial flight simulator has three rotational frames, namely, the outer frame, middle frame, and inner frame. The controlling flight simulator was achieved by setting different configurations in the computer software.

As can be seen from Figure 10, the RSSINS is firmly installed on the tri-axial flight simulator though a fixed structure. Different rotation schemes, such as rotation about the *X*-axes, *Y*-axes, and Z-axes, can be implemented by rotating the three frames [32]. For example, with the IMU axis defined as shown in Figure 10, the inner frame rotates around the *X*-axis to make a roll motion, the middle frame rotates around the *Z*-axis to make a pitch motion, and the outer frame rotates around the *Y*-axis to make a yaw motion.

Table 2 and Table 3 summarize the technical parameters of the tri-axial flight simulator and the characteristics of the sensors in the RSSINS, respectively.

To verify the effectiveness of the new rotation scheme for residual error suppression in the navigation system, the experiments will be carried out in an actual system and compared to the standard information provided by the tri-axial flight simulator. The RSSINS was installed on the turntable, with the IMU sensitive axes defined as the *X*-axis pointing forward, the *Z*-axis pointing right, and the *Y*-axis pointing up, with the rotation of the inner frame rotating the system about its *X*-axis. Table 4 shows the settings of the experimental conditions. The specific experiment conditions were set as follows:

Experiment 1: The inner frame was rotated at high speed (9000°/s) to simulate the high-rotation motion of the missile for the system. The middle and outer frames of the turntable simulated the pitch and yaw motion of the missile. The RSSINS was installed on the turntable and the dual function of rotation reduction and rotation modulation was realized by the rotary mechanism. In RSSINS, the preset rotation scheme was single axis continuous rotation.

Experiment 2: The MIMU was directly installed on the turntable and the ideal modulation angular rate was provided by the inner frame of the turntable. The preset rotation scheme was a single axis continuous rotation. Similarly, the middle and outer frames of the turntable simulated the pitch and yaw motion of the missile.

Experiment 3: The setting of the turntable was the same as in Experiment 1. The RSSINS was installed on the turntable and the preset rotation scheme was a single axis continuous reciprocating rotation.

Experiment 4: The setting of the turntable was the same as in Experiment 2. The MIMU was installed on the turntable and the preset rotation scheme was a single axis continuous reciprocating rotation.

Experiment 5: The setting of the turntable was the same as in Experiment 1. The RSSINS was installed on the turntable and the preset rotation scheme was the new rotation scheme.

Experiment 6: The setting of the turntable was the same as in Experiment 2. The MIMU was installed on the turntable and the preset rotation scheme was the new rotation scheme.

**Figure 11:**
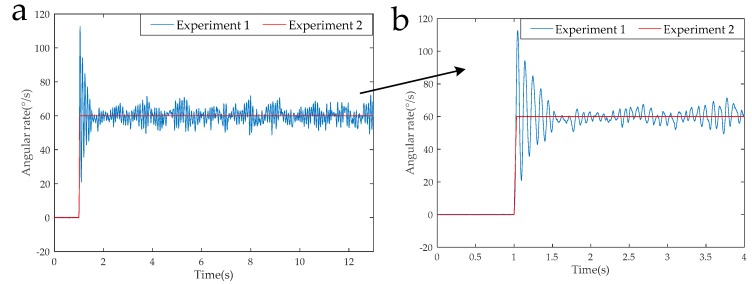
(**a**) Modulation angular rates of Experiment 1 and Experiment 2. (**b**) THE partial enlarged view of modulation angular rates.

**Figure 12:**
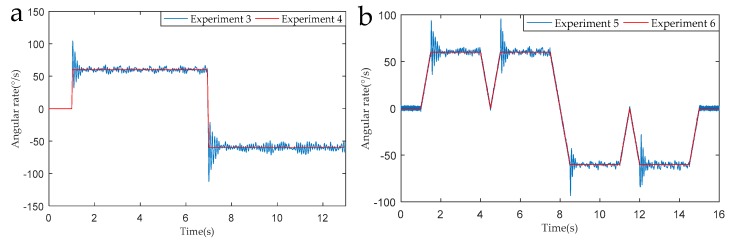
(**a**) Modulation angular rates of Experiment 3 and Experiment 4. (**b**) Modulation angular rate of Experiment 5 and Experiment 6.

Figure 11 shows the modulation angular rate of Experiment 1 and Experiment 2 and Figure 12 shows the modulation angular rate of Experiments 3 and 4, and Experiments 5 and 6. The experimental implementation detail is as follows: Experiments 1 and 2, and 3 and 4 are two different rotation schemes, as shown in Table 3, and the specific rotation process is shown in Figure 7 and Figure 8. The specific rotation process of Experiments 5 and 6 is shown in Figure 9. The difference is that the new modulation scheme sets acceleration and deceleration with an angular acceleration of 2/3π rad/s^2^.

It can be seen from the partial enlarged view of Figure 11 that the actual angular rate provided by the rotary mechanism has an acceleration-deceleration process and it has poor stability and overshoot during angular rate changes. We can know from Figure 12, in the RSSINS, compared to the new rotation scheme (Experiment 5), that the angular rate provided by the rotary mechanism in the single axis continuous positive and negative rotation scheme (Experiment 3) has a larger overshoot and modulation angular rate error. This is because the rapidity and stability of the motor cannot be achieved at the same time. In addition, there is no sudden change in the modulation angular rate of the new rotation scheme, so the angular rate error is reduced.

**Figure 13:**
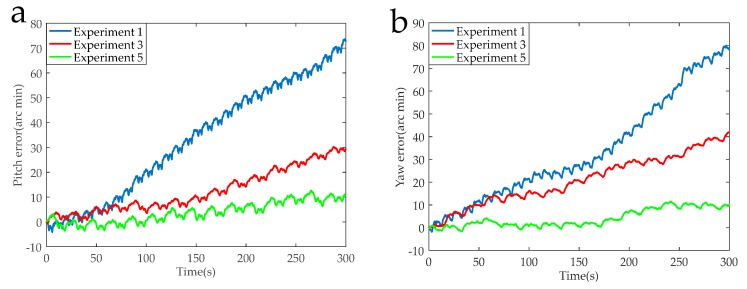
(**a**) Pitch error curves of the Experiments 1, 3, and 5. (**b**) Yaw error curves of the Experiments 1, 3, and 5.

**Figure 14:**
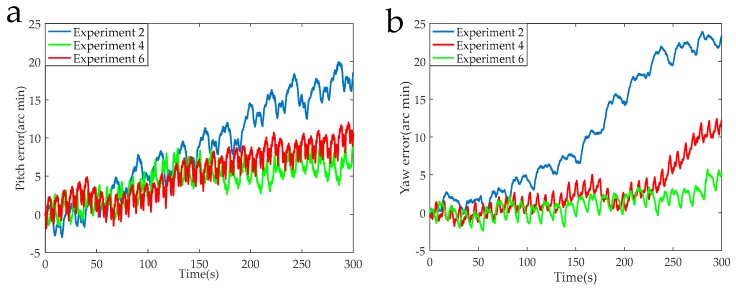
(**a**) Pitch error curves of the Experiments 2, 4, and 6. (**b**) Yaw error curves of the Experiments 2, 4, and 6.

**Figure 15:**
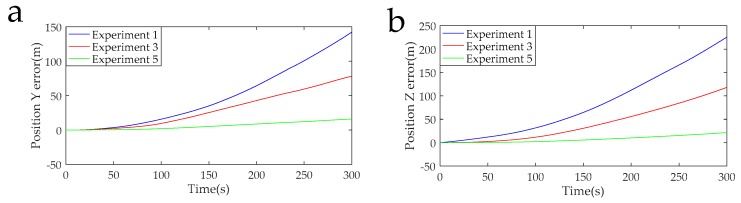
(**a**) Position Y error curves of the Experiments 1, 3, and 5. (**b**) Position Z error curves of the Experiments 1, 3, and 5.

**Figure 16:**
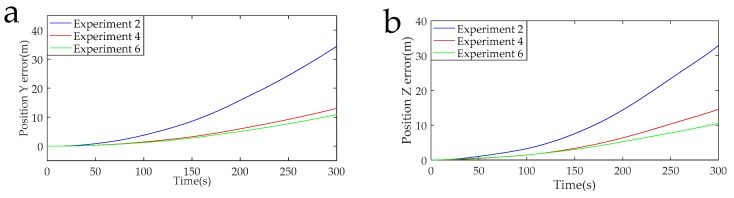
(**a**) Position Y error curves of the Experiments 2, 4, and 6. (**b**) Position Z error curves of the Experiments 2, 4, and 6.

The data acquisition circuit is known to have a sampling rate of 5 K, and 1,500,000 points were intercepted for navigation solution. The calculated navigation information in the RSSINS and in the MIMU were compared with the standard information provided by the turntable, and then the attitude and position errors were obtained, as shown in Figure 13, Figure 14, Figure 15 and Figure 16. Table 5 is the maximum value of the attitude and position error of the different rotation schemes.

By specifically analyzing the experimental data of the different rotation schemes given in Table 4, the following conclusions can be obtained.

In the actual RSSINS, the pitch angle error reduces from 73.3′ in Experiment 1 to 10.9′ in Experiment 5, and the yaw angle error reduces from 80.0′ in Experiment 1 to 10.2′ in Experiment 5. The reason for this is that in the continuous rotation scheme, the angular rate provided by the rotating mechanism changes only once, so the dynamic error of the angular velocity is relatively small, but the scale factor error is coupled with the rotational speed, resulting in a large magnitude error, which affects the modulation effect. In the actual system, the pitch angle error reduces from 17.1′ in Experiment 3 to 10.9′ in Experiment 5, and the yaw angle error reduces from 41.2′ in Experiment 3 to 10.2′ in Experiment 5. This is because, in the reciprocation rotation scheme, the angular rate changes frequently, and the residual error due to the angular rate error accumulates over time. By comparing Experiments 1, 3, and 5, in the actual system, we can conclude that the navigation accuracy of the new rotation scheme has increased by at least 56% compared to the existing rotation scheme.

When the modulation angular rate is provided by a high-precision turntable, Experiment 4 has a pitch angle error of 8.8′, which is similar to the pitch angle error of 9.3′ in Experiment 6. Moreover, the yaw angle error and the position error are similar. This comparison verifies that the angular velocity error of the rotating mechanism greatly affects the navigation accuracy. Therefore, it is necessary to design a new rotation scheme to compensate for the modulation angular rate error in a high-dynamic environment.

Based on the results from the experiments, we can see that with the limited size and cost of the rotating mechanism, the new rotation scheme helps to improve the navigation accuracy of low-cost MEMS inertial sensors.

## 7. Conclusions

In this paper, first of all, the impact of the rotating mechanism’s angular rate error on the navigation solution was investigated. Then according to the characteristics of the actual output angular rate of the rotating mechanism, a novel modulation scheme for an RSSINS in a high-rotation missile-borne environment was proposed for the first time. This scheme makes it possible to eliminate the constant drift’s residual error in the acceleration-deceleration process and the angular rate instability error by setting a symmetrical motion state at a symmetrical position. Additionally, there is no sudden change in the angular rate in the new scheme, which solves the contradiction between the rapidity and stability of the motor speed output. In addition, the navigation accuracy of the MEMS sensor under high dynamic conditions is improved without relying on software or other navigation methods. Finally, by carrying out several contrast experiments, experiment results showed that the position and attitude accuracy of the new rotation scheme is increased by more than 56%. The next step is to comprehensively consider the navigation error model and the error model of the motor’s actual output angular rate in the RSSINS in order to find the optimal acceleration and angular rates that can achieve higher navigation accuracy.

## Figures and Tables

**Table 1:** Constant drift modulation effect after one cycle of different rotation schemes.

Drift after a Complete Rotation Cycle	Ideal Reciprocating Rotation	Considering Motor Error Reciprocating Rotation	**Considering Motor Error New Rotation Scheme**
Residual error during acceleration and deceleration	$\left[\begin{array}{l} T\varepsilon_{z}^{s}\\ 0\\ 0 \end{array}\right]$	$\left[\begin{array}{c} \begin{array}{l} T\varepsilon_{z}^{s} \end{array}\\ \frac{2\theta_{a}-\sin\theta_{a}}{\pi }T\varepsilon_{y}^{s}\\ \frac{2\theta_{a}-\sin\theta_{a}}{\pi }T\varepsilon_{z}^{s} \end{array}\right]$	$\left[\begin{array}{l} T\varepsilon_{z}^{s}\\ 0\\ 0 \end{array}\right]$
Angular rate instability	$\left[\begin{array}{l} T\varepsilon_{z}^{s}\\ 0\\ 0 \end{array}\right]$	$\left[\begin{array}{c} \begin{array}{l} T\cdot \varepsilon_{x} \end{array}\\ \frac{2\varepsilon_{y}\cdot \sin\left(\frac{2\pi \cdot \delta \omega }{\omega }\right)}{\omega +\delta \omega }\\ \frac{2\varepsilon_{z}\cdot \sin\left(\frac{2\pi \cdot \delta \omega }{\omega }\right)}{\omega +\delta \omega } \end{array}\right]$	$\left[\begin{array}{l} T\varepsilon_{z}^{s}\\ 0\\ 0 \end{array}\right]$

**Table 2:** Technical parameters of the tri-axial flight simulator.

Position Accuracy (°)	Rotation Rate (°/s)		
	Inner Frame	Middle Frame	Outer Frame
0.001	0.001–12,000	0.001–400	0.001–400

**Table 3:** Parameters of the sensors in RSSINS.

Characteristics	Range	Bias	Random Walk
Gyroscope (*X*-axis)	±200°/s	20°/h	$0.6\;{}^{\circ}/\sqrt{Hz}$
Gyroscopes (*Y*-, *Z*-axis)	±75°/s	12°/h	$0.28\;{}^{\circ}/\sqrt{Hz}$
Accelerometer (*X*-axis)	±10 g	0.75 mg	150 µg/$\sqrt{Hz}$
Accelerometer (*Y*-, *Z*-axis)	±2.5 g	0.75 mg	100 µg/$\sqrt{Hz}$
Optical encoder	Resolution	360°/1024	—

**Table 4:** Settings of the experiment conditions.

	Pitch	Yaw	MIMU Roll Angular Rate	Rotating Mechanism	Rotating Scheme
Experiment 1	+30°~−30°	0°	60°/s	The RSSINS	Single axis continuous rotation
Experiment 2	+30°~−30°	0°	60°/s	High-precision turntable	Single axis continuous rotation
Experiment 3	+30°~−30°	0°	60°/s	The RSSINS	Single axis continuous reciprocating rotation
Experiment 4	+30°~−30°	0°	60°/s	High-precision turntable	Single axis continuous reciprocating rotation
Experiment 5	+30°~−30°	0°	60°/s	The RSSINS	New rotation scheme
Experiment 6	+30°~−30°	0°	60°/s	High-precision turntable	New rotation scheme

**Table 5:** Maximum error in position and attitude of different rotation schemes.

	Pitch Error (′)	Yaw Error (′)	Position *Y* Error (m)	Position *Z* Error (m)
Experiment 1	73.3	80.0	141.4	224.2
Experiment 2	18.1	23.3	33.9	32.9
Experiment 3	17.1	41.2	77.5	116.8
Experiment 4	8.8	12.1	12.9	14.4
Experiment 5	10.9	10.2	15.9	20.9
Experiment 6	9.3	5.4	10.8	10.5
